# Use of monoclonal antibody therapy in hematologic patients with mild‐to‐moderate COVID‐19: A retrospective single‐center experience

**DOI:** 10.1002/cam4.5832

**Published:** 2023-04-20

**Authors:** Idoroenyi Amanam, Janny Yao, Alfredo Puing, Ni‐Chun Tsai, Diana Samuels, Dat Ngo, Stephanie Ho, Haris Ali, Ahmed Aribi, Shukaib Arslan, Andrew Artz, Myo Htut, Paul Koller, Amandeep Salhotra, Karamjeet Sandhu, Liana Nikolaenko, Anna Pawlowska, Geoffrey Shouse, Anthony Stein, Guido Marcucci, Stephen Forman, Ryotaro Nakamura, Sanjeet Dadwal, Monzr M. Al Malki

**Affiliations:** ^1^ Department of Hematology and Hematopoietic Cell Transplantation City of Hope National Medical Center Duarte California USA; ^2^ Department of Pharmacy City of Hope National Medical Center Duarte California USA; ^3^ Department of Infectious Diseases City of Hope National Medical Center Duarte California USA; ^4^ Department of Computational and Quantitative Medicine, Division of Biostatistics City of Hope National Medical Center Duarte California USA; ^5^ Department of Pediatrics City of Hope National Medical Center Duarte California USA

**Keywords:** cancer stem cells, hematologic malignancies, leukemia, target therapy, viral infection

## Abstract

**Introduction:**

In November 2020, the FDA issued an emergency use authorization (EUA) for monoclonal antibody (mAb) therapy in patients with mild‐to‐moderate COVID‐19 at high risk for disease progression.

**Methods:**

We retrospectively reviewed 38 adult hematology patients who received mAbs from 11/2020 to 2/2021.

**Results:**

Thirty (79%) patients received bamlanivimab and 8 (21%) casirivimab‐imdevimab. Four (11%) patients were hospitalized due to COVID‐19, two (5%) progressed to severe disease and one patient (3%) died within 30 days from COVID‐19 disease. Most patients (*n* = 34, 89%) ultimately tested negative for SARS‐CoV‐2, with 34% (*n* = 13) clearing the virus within 14 days after mAb infusion. The median time to clearance of viral shedding was 25.5 days (range: 7–138). After mAb infusion, most patients with hematological malignancies (HM) (*n* = 10/15; 67%) resumed therapy for underlying disease with a median delay of 21.5 days (range: 12–42). We observed a significant difference in hospitalization among patients who received a HCT versus non‐HCT (0% *n* = 0/26 and 36% *n* = 4/11, respectively; *p* < 0.01).

**Conclusions:**

This study demonstrates that SARS‐CoV‐2 specific mAb was safe and may reduce hospitalization compared to what is reported in malignant hematology patients at high risk for disease progression. Our HCT cohort patients had less hospitalization rate compared with HM cohort patients.

## Graphical abstract

Presenting symptoms noted at the time of COVID‐19 diagnosis.

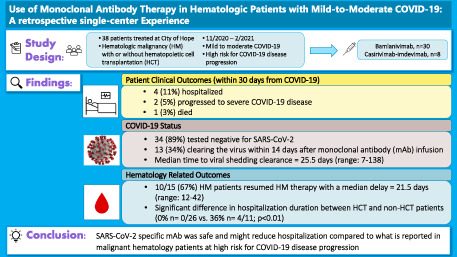

**Key points**

- SARS‐CoV‐2 monoclonal antibody therapy in malignant hematology patients was safe and may reduce hospitalization.
- The access to SARS‐CoV‐2 monoclonal antibodies is important in preventing progression to severe COVID‐19 disease and hospitalization.

## 1 INTRODUCTION

Severe acute respiratory syndrome coronavirus 2 (SARS‐CoV‐2) is a novel coronavirus that causes coronavirus disease 2019 (COVID‐19), and the cause of global pandemic which has infected hundreds of millions and killed over six million people worldwide.^1^

The presentation of COVID‐19 is diverse, ranging from asymptomatic patients who only have a positive SARS‐CoV‐2 test versus those who progress to critical illness with multiorgan failure. Approximately 80% of patients who test positive for SARS‐CoV‐2 will present with mild‐to‐moderate disease.^2^ SARS‐CoV‐2 viral replication occurs before the onset of symptoms and diminishes days after symptoms have begun. From our experience with other viral infections, it is thought that the antivirals will exert their benefit in the viral replicative phase, which occurs early on.

SARS‐CoV‐2 enters the respiratory cells by binding its spike protein to the angiotensin converting enzyme 2 (ACE2) receptor. Potent antispike neutralizing monoclonal antibodies (mAb) have a high affinity to the receptor‐binding domain of spike protein of SARS‐CoV‐2 and reduce the attachment of the spike protein with the human ACE2 receptor.^3^ Therefore, monoclonal antibodies reduce viral replication, leading to clinical improvement and preventing hospitalization.

In November 2020, the US Food and Drug Administration (FDA) issued an emergency use authorization (EUA) for mAb therapy in patients with mild‐to‐moderate COVID‐19 who are at high risk for disease progression. These mAbs reduce the risk of hospitalization in the general population. However, its efficacy and safety in immunocompromised hematology patients is not well described.

This study aims to assess the impact of mAb in nonhospitalized patients with mild‐to‐moderate COVID‐19 undergoing treatment for hematological malignancies (HM) at City of Hope National Medical Center. Our outcomes included rate of hospitalization, time to clearance of infection, HM treatment delay, and 30‐day mortality.

## 2 METHODS

This study was approved by the City of Hope's (COH) Institutional Review Board. The EUA for mAb is limited for the treatment of patients with mild‐to‐moderate COVID‐19 who are at high risk for severe disease or hospitalization. The EUA defines high risk as patients meeting at least one of the following criteria: body mass index (BMI) ≥35, chronic kidney disease (CKD), diabetes, immunosuppressive disease or receiving immunosuppressive treatment, ≥65 years of age or ≥55 years of age and have cardiovascular disease (CVD), hypertension, or chronic obstructive pulmonary disease (COPD).^4^, ^5^ We retrospectively reviewed a case series of 38 adult hematology patients with mild‐to‐moderate COVID‐19 disease who received monoclonal antibodies within 10 days of symptoms onset from November 9th, 2020, until February 28th, 2021. Patients received either a single infusion of bamlanivimab 700 mg or casirivimab‐imdevimab 600/600 mg. Patients who were asymptomatic, had severe or critical COVID‐19 disease, or were hospitalized at the time of COVID‐19 diagnosis were excluded. Baseline demographic, clinical outcomes, and hematologic‐related data were extracted. All statistical analysis was performed using SAS statistical software.

## 3 RESULTS

### 3.1 Demographics, Disease, and Treatment Characteristics

Thirty‐eight HM patients with mild‐to‐moderate COVID‐19 disease who received mAb therapy under EUA were included in this study. Thirty (79%) patients received bamlanivimab and 8 (21%) patients received casirivimab‐imdevimab. Baseline characteristics prior to mAb administration include: 53% female, median age of 51 years (range: 21–80), with 18% above 65 years old. Eighteen (7%) patients had a BMI ≥35 with a median BMI of 27. Six patients (16%) had CKD; six patients (16%) had diabetes; 17 (45%) patients had hypertension; five (13%) patients had CVD; 10 (38%) patients were former smokers; and six patients (16%) had prior lung disease. Fifteen (39%) patients had lymphopenia (ALC ≤1000). The median number of risk factors for COVID‐19 disease progression or hospitalization was two (range 1–4). (Table 1).

**TABLE 1:** Demographics and disease characteristics.

Variable	All patients (*n* = 38) *N* (%) or median (range)
Patient's Age	51 (21–80)
Gender	
Female	20 (53)
Male	18 (47)
Race/ethnicity	
Hispanic	14 (37)
Asian	5 (13)
Black	4 (11)
White	15 (39)
Primary disease	
AML	12 (31)
ALL	6 (16)
Lymphoma	8 (21)
MM	8 (21)
CLL/AA	4 (11)
Cellular therapy	
Allogeneic	18 (47)
Autologous	9 (24)
CAR‐T	1 (3)
None	10 (26)
ISI score	3 (0–6)
Number of prior therapy	3 (1–6)
BMI	27.0 (16.4–48.9)
BMI ≥35	18 (7)
Chronic kidney disease	
Yes	6 (16)
No	32 (84)
Diabetes	
Yes	6 (16)
No	32 (84)
Hypertension	
Yes	17 (45)
No	21 (55)
Cardiovascular disease	
Yes	5 (13)
No	33 (87)
Former smoker	
Yes	10 (38)
No	28 (62)
Lung disease	
Yes	6 (16)
No	32 (84)
Risk factors for progressing to severe COVID‐19 and/or hospitalization	2 (1–4)
Outcomes after mAb	
Hospitalization	
Yes	4 (11)
No	34 (89)
Days from mAb infusion to hospitalization	7.5 (0–17)

Use of monoclonal antibody therapy in hematologic patients with mild‐to‐moderate COVID‐19: A retrospective single‐center experience

Twenty‐eight (74%) patients received cellular therapy: 18 (47%) had undergone allogeneic hematopoietic cell transplantation (HCT), nine (24%) autologous HCT, and one (3%) chimeric antigen receptor T cell (CAR T) therapy. Median time from cellular therapy to diagnosis of COVID‐19 was 409 days (range: 182–4692). Ten patients (26%) had treatment delays due to COVID‐19 with a median time of 21 days.

Among the 17 patients who had COVID‐19 disease after allogeneic HCT (alloHCT), the median time to COVID‐19 diagnosis from HCT was 22.8 months (range: 2.6–274.4). Twelve out of 17 (71%) alloHCT patients were managed for active graft‐versus‐host disease (GvHD) at the time of COVID‐19 diagnosis (chronic GvHD: n = 11 [mild: 4, moderate: 4, and severe: 3], acute GvHD: n = 1 [grade 2]). Three out of 12 patients (25%) were on systematic steroids while being managed for GvHD. Ten (59%) alloHCT patients were on immunosuppressant therapy at the time of COVID‐19 diagnosis. Fifteen (39%) patients were on active treatment for their HM at the time of COVID‐19 diagnosis with a mean of three previous lines of treatment (range: 1–6). Additional patient characteristics are shown in Table 1.

### 3.2 COVID‐19, Hospitalization

At the time of COVID‐19 diagnosis, the most common presenting symptoms included: cough, fever, headache, fatigue, and body aches. (Table 2). Four (11%) patients were hospitalized due to COVID‐19, and two (5%) progressed to severe disease. All four patients had received bamlanivimab three out of four patients had an ALC ≤1000. The median time to hospitalization from COVID‐19 diagnosis was 8 days (range: 1–20), while the median time from mAb infusion to hospitalization was 7.5 days (range: 0–17). Two patients (5%) received remdesivir subsequently. One patient (3%) was admitted to ICU who succumbed to COVID‐19 and died within 30 days of COVID‐19 diagnosis.

**TABLE 2:** Symptoms at diagnosis.

Variable	All patients (*n* = 38) *N* (%)
Cough	22 (58)
Fever	14 (37)
Headache	13 (34)
Fatigue	11 (29)
Body aches	11 (29)
Sore throat	9 (24)
Loss of taste	8 (21)
Chills	7 (18)
Loss of smell	7 (18)
Nausea	4 (11)
Loss of appetite	3 (8)

We observed a significant difference in hospitalization rate between HCT recipients and nonrecipients (0%, *n* = 0/26 and 36%, *n* = 4/11, respectively; *p* < 0.01). None of the other patient characteristics, which included: gender, ethnicity, age, BMI, smoking, obesity, chronic kidney disease, diabetes mellitus, hypertension, coronary vascular disease, and lung disease, were associated with a significantly increased hospitalization rate.

### 3.3 Viral clearance and safety

Most patients (*n* = 34, 89%) ultimately tested negative for SARS‐CoV‐2 by PCR after mAb infusion. 34% of patients (*n* = 13) cleared the virus within 14 days of receiving mAb infusion. The median time to clearance of viral shedding was 25.5 days (range: 7–138). After mAb infusion, most patients (10/15; 67%) who were previously on active treatment for HM prior to diagnosis of COVID‐19 resumed therapy for their HM with a median delay of 21.5 days (range: 12–42). mAb therapy under EUA was well tolerated in this patient population with only one (3%) patient having experienced an adverse reaction characterized as headache.

## 4 DISCUSSION

Herein we describe our experience using mAb to treat nonhospitalized patients with mild‐to‐moderate COVID‐19 undergoing treatment for hematologic malignancies with or without HCT.

In the studies that supported the EUA from the FDA for casirivimab‐Imdevimab and bamlanivimab, only 4% and 34% of patients were considered at high risk, respectively.^4^, ^6^ By contrast, all patients in our cohort were at high risk for progressing to severe COVID‐19 and/or hospitalization.

Our patient cohort's clinical characteristics and presentation were comparatively different than those published in patients with COVID‐19 who had hematologic malignancies. Our population was younger (82% under 65) than those published by ASH, EHA, and Israel and similar to those cohorts that were presented under the EUA for casirivimab‐imdevimab and bamlanivimab.^7^, ^8^, ^9^

The overall hospitalization rate was 11% in our cohort. This was higher than the reported hospitalization of 1.6% in bamlanivimab and 3% in high‐risk casirivimab‐imdevimab cohorts of the respective studies in the general population.^4^, ^6^ However, this was much lower than the reported 73% hospitalization rate in EHA samples of 3801 HM patients.^8^ Interestingly, our HCT cohort had 0% hospitalization compared with HM cohort. In our cohort ICU care was required by 3% of patients, which is lower than reported in hematological malignancies.^8^ This observation is most likely related to early identification of infection and treatment with mAb, thereby preventing progression to lower respiratory tract disease/severe COVID‐19 disease.

The one mortality in our cohort had a diagnosis of mantle cell lymphoma. This patient had relapsed disease and was treated with anti‐CD20 monoclonal antibody rituximab and the Bruton tyrosine kinase (BTK) inhibitor ibrutinib. Patients treated with BTK inhibitors have high rates of infectious complications, including pneumonia, even with preserved CD‐4 cell counts.

Lymphopenia (defined as ALC <200) has been identified as an independent risk factor for progression of community respiratory viruses (CRV) such as respiratory syncytial virus (RSV), influenza virus from upper to lower respiratory tract.^10^ Lymphopenia has been identified as a feature of COVID‐19 but in patients with impaired adaptive immunity it is unclear what role lymphopenia plays in the severity of COVID‐19 disease.^11^ Fifteen (39%) patients had lymphopenia (ALC ≤1000) but there was no definitive signal regarding lymphopenia and outcomes. Furthermore, the institution of mAb may lead to a beneficial effect in preventing progression when given early in the course of illness with SARS‐CoV‐2.

The limitations of our study include those of retrospective studies, changing therapeutic landscape as the pandemic evolved over time, access to monoclonal antibodies based on their availability from local health departments and using in patients at highest risk for severe COVID19 disease. However, the latter does not seem to have to negatively impact the benefit of mAb. We were unable to carry out immune subset assessment to determine in a more specific way the degree of immune compromise. In addition, we did not have the variant information available for our patients, though for the time period, the state of California had Epsilon as the predominant variant.^12^

Even though with the evolving SARS‐CoV2 variants have upended the efficacy of many of the EUA mAbs, and taken off from clinical use, our study describes the use of monoclonal antibodies and their safety in patients with hematologic malignancies which has been demonstrated in other studies and case series.^13^, ^14^

In conclusion, this study demonstrates that SARS‐CoV2 specific mAb use in malignant hematology patients under EUA was safe and may reduce hospitalization as reported in the literature among those at high risk for disease progression. Thus, the access to SARS‐CoV2 mAb in this population which is at increased risk for complications from SARS‐CoV2 infection is critical in reducing the progression to severe COVID‐19 disease and hospitalization.

## AUTHOR CONTRIBUTIONS

**Idoroenyi Amanam:** Conceptualization (equal); data curation (equal); formal analysis (equal); investigation (equal); methodology (equal); writing – original draft (equal); writing – review and editing (equal). **Janny Yao:** Conceptualization (equal); data curation (equal); formal analysis (equal); investigation (equal); methodology (equal); writing – original draft (equal); writing – review and editing (equal). **Alfredo Puing:** Writing – original draft (equal); writing – review and editing (equal). **Ni‐Chun Tsai:** Formal analysis (supporting); methodology (supporting). **Diana Samuels:** Writing – review and editing (supporting). **Dat Ngo:** Writing – review and editing (supporting). **Stephanie Ho:** Writing – review and editing (supporting). **Haris Ali:** Writing – review and editing (supporting). **Ahmed Aribi:** Writing – review and editing (supporting). **Shukaib Arslan:** Writing – review and editing (supporting). **Andrew Artz:** Writing – review and editing (supporting). **Myo Htut:** Writing – review and editing (supporting). **Paul Koller:** Writing – review and editing (supporting). **Amandeep Salhotra:** Writing – review and editing (supporting). **Karamjeet Sandhu:** Writing – review and editing (supporting). **Liana Nikolaenko:** Writing – review and editing (supporting). **Anna Pawlowska:** Writing – review and editing (supporting). **Geoffrey Shouse:** Writing – review and editing (supporting). **Anthony Stein:** Writing – review and editing (supporting). **Guido Marcucci:** Writing – review and editing (supporting). **Stephen Forman:** Writing – review and editing (supporting). **Ryotaro Nakamura:** Writing – review and editing (supporting). **Sanjeet Dadwal:** Formal analysis (supporting); investigation (supporting); methodology (supporting); writing – original draft (supporting); writing – review and editing (supporting). **Monzr M. Al Malki:** Data curation (supporting); methodology (supporting); project administration (supporting); writing – original draft (supporting); writing – review and editing (supporting).

## CONFLICT OF INTEREST STATEMENT

The authors have no relevant conflicts of interest to declare.
